# P22. Immunomodulation induced by resveratrol or genistein on proliferation and apoptosis of tumour colon cells

**DOI:** 10.1186/2051-1426-2-S2-P13

**Published:** 2014-03-12

**Authors:** L Brasoveanu, C Hotnog, M Bostan, G Matei, D Hotnog, M Gruia, M Mihaila

**Affiliations:** 1 Center of Immunology Inst."Stefan S.Nicolau", Tumour Immunology, Bucharest, Romania; 2 "Prof. Dr. Alex. Trestioreanu" Institute of Oncology, Cancer Biochemistry, Bucharest, Romania

## 

Colon cancer represents a malignancy with a high incidence and mortality throughout the world, its etiology involving many genetic, immunological and biochemical factors. Anti-cancer drugs might exert their action during physiological pathways of apoptosis, leading to tumour cell destruction. One of the most effective anti-cancer agents used in the treatment of colorectal cancers is 5-fluorouracyl (5-FU), but tumour chemoresistance is a major limiting factor of its use. The purpose of this study was to assess whether different doses of plant bioactive compounds as genistein (GST) or resveratrol (RSV) might increase the anti-carcinogenic and anti-proliferative effects of 5-FU treatment and modulate gene *vs.* antigen expression of molecules involved in cell proliferation and apoptosis (P53, Bcl-2, Bax, Mdm2, PTEN) in colon tumour cell lines (LoVo).

The compound-mediated cytotoxity was measured by real-time cell analysis (RTCA) using xCELLigence System, a cell-based label-free platform technology that utilises the inherent morphological and adhesive characteristics of the cell. DNA progression through cell cycle phases was estimated by using PI staining (BD Cycletest Plus/DNA Reagent kit), while apoptosis was assessed by Annexin-V/FITC and PI double staining. In addition, antigen expression was evaluated by indirect immunofluorescence followed by flow-cytometry acquisition and analysis by FACS Canto II flow-cytometer. Gene expression was measured by qRT-PCR using Applied Biosystems® 7300 System.

Both GST and RSV single treatments, or combined with 5-FU induced a decrease of S% cell cycle phase, and an increse of apoptotic events. Different concentrations of GST or RSV blocked the treated cells in G2M; cells treated with RSV and 5-FU were blocked in G0G1 phase, while combined treatments with GST and 5-FU blocked the cells in G2M phase. The gene and antigen expression of molecules associated to cell proliferation and apoptosis were modulated by single and combined treatments, and additive effects of RSV or GST to 5-FU treatment were observed.

The chemo-preventive efficacy has been associated to enhanced apoptosis, therefore any therapeutic strategy that specifically triggers apoptosis in cancer cells might have potential immunotherapeutic value. By combining flavonoids with anti-cancer drugs, an increase of the antitumoral effects might be achieved, specifically in highly invasive cancer cells, while in non-tumoral cells the cytotoxic side effects could be reduced.

